# Full Mitogenomes in the Critically Endangered Kākāpō Reveal Major Post-Glacial and Anthropogenic Effects on Neutral Genetic Diversity

**DOI:** 10.3390/genes9040220

**Published:** 2018-04-19

**Authors:** Nicolas Dussex, Johanna von Seth, Bruce C. Robertson, Love Dalén

**Affiliations:** 1 Department of Bioinformatics and Genetics, Swedish Museum of Natural History, SE-10405 Stockholm, Sweden; johanna.vonseth@nrm.se (J.v.S.); Love.Dalen@nrm.se (L.D.); 2 Department of Zoology, University of Otago, Great King Street, Dunedin 9016, New Zealand; bruce.robertson@otago.ac.nz; 3 Department of Zoology, Stockholm University, SE-10691 Stockholm, Sweden

**Keywords:** bottleneck, expansion, mitogenome, endemic, historical DNA, genetic diversity

## Abstract

Understanding how species respond to population declines is a central question in conservation and evolutionary biology. Population declines are often associated with loss of genetic diversity, inbreeding and accumulation of deleterious mutations, which can lead to a reduction in fitness and subsequently contribute to extinction. Using temporal approaches can help us understand the effects of population declines on genetic diversity in real time. Sequencing pre-decline as well as post-decline mitogenomes representing all the remaining mitochondrial diversity, we estimated the loss of genetic diversity in the critically endangered kākāpō (*Strigops habroptilus*). We detected a signal of population expansion coinciding with the end of the Pleistocene last glacial maximum (LGM). Also, we found some evidence for northern and southern lineages, supporting the hypothesis that the species may have been restricted to isolated northern and southern refugia during the LGM. We observed an important loss of neutral genetic diversity associated with European settlement in New Zealand but we could not exclude a population decline associated with Polynesian settlement in New Zealand. However, we did not find evidence for fixation of deleterious mutations. We argue that despite high pre-decline genetic diversity, a rapid and range-wide decline combined with the lek mating system, and life-history traits of kākāpō contributed to a rapid loss of genetic diversity following severe population declines.

## 1. Introduction

Significant attention has been devoted to examining the effects of extrinsic processes such as habitat modification, over-hunting or persecution on species persistence. However, there is still a gap in our understanding on how intrinsic genetic processes contribute to extinctions [1,2,3]. Theory predicts that small and isolated populations will lose genetic diversity, become exposed to the effects of inbreeding and accumulate deleterious mutations. The resulting reduction in fitness might contribute to some extent to the eventual extinction of species [4,5]. In fact, recent studies of extinct species have shown an increase in inbreeding [3] and accumulation in deleterious mutations in both nuclear [6] and mitochondrial DNA [7] immediately before their extinction, suggesting that geographical isolation and the associated effects of genetic drift and inbreeding may have contributed to the extinction event.

In the current 6th mass extinction [8], it is crucial to understand how these intrinsic genetic processes affect species extinction risk because many species are now surviving in the form of fragmented populations and often show important reduction in genetic diversity. As a case in point, the critically endangered kākāpō (*Strigops habroptilus*), an endemic flightless parrot of New Zealand, has gone through a severe population decline since human arrival and in particular in the last 200 years [9]. Despite being one of the most abundant avian species in New Zealand upon European arrival [10], kākāpō currently number 150 birds [11], all descending from two isolated populations. Fossil deposits suggest that kākāpō were widespread and abundant throughout the North and South Islands [12,13,14,15,16,17,18] of New Zealand. However, while kākāpō were present on Stewart Island as evidenced by Māori midden remains [19], fossil remains have not been found, either suggesting a possible human introduction [19] or low fossil preservation due the acidic soil [9,10].

The species’ range contracted following Polynesian arrival c. 1280 CE [20], through the combined effects of habitat modification by burning and hunting [10,15]. However, early observations suggest kākāpō remained locally abundant in less intensively settled areas up to the 19th century until the introduction of mammalian predators by European settlers [19,21]. While Polynesian hunting may also have impacted kākāpō prior to European arrival, previous microsatellite and mitochondrial data did not support a Polynesian-driven bottleneck in South Island kākāpō [22]. Kākāpō were likely extirpated from the North Island in the early 20th century [19] and by the 1970s, the species was reduced to a few males in the Fiordland (southwest New Zealand; [9,23]) and a small population on Stewart Island (30 km south of the South Island [24]).

The current 150 extant kākāpō are actively managed on offshore island sanctuaries [25]. This population includes some of the original 61 founders of the sanctuary population translocated in the 1970s and their descendants. While this founding population has increased since the 1980s, there is strong evidence for inbreeding depression including poor sperm quality and low hatching success [26].

Here we generated high-quality mitogenomes for 39 historical (1800s–1900s) and 79 modern kākāpō representing all the extant mitochondrial diversity to (1) quantify the loss of genetic variation associated with human settlement in New Zealand and (2) to examine population fluctuations in relation to post-glacial expansion and anthropogenic effects.

## 2. Materials and Methods

### 2.1. Sampling

39 kākāpō historical skin specimens collected between 1847 and 1985 from the South Island of New Zealand and representing the pre-European population were sourced from various museum collections (Table S1). These 39 samples represent a subset of the 54 samples used in Bergner et al. [22]. Also, 79 blood samples from birds surviving the population bottleneck (i.e., founders of the extant managed population, *n* = 42) and some of their offspring (F1s, *n* = 33; F2s, *n* = 4) were provided by the New Zealand Department of Conservation (DOC).

### 2.2. DNA Extractions and DNA Library Preparation

Genomic DNA was extracted from historical skins using a DNeasy Blood & Tissue Kit (Qiagen, Hilden, Germany). Appropriate precautions were taken to minimize the risk of contamination in historical samples [27]. For contemporary samples, genomic DNA was extracted from blood using a phenol-chloroform protocol.

For historical specimens, double stranded Illumina libraries were prepared according to Meyer & Kircher [28] using uracil-treatment with the USER enzyme (New England Biolabs, Ipswich, MA, USA) and indexing PCRs with AccuPrimeTM Pfx DNA Polymerase (Life Technologies, Carlsbad, CA, USA). Indexing amplifications were prepared using one indexing primer per library. Purification and size selection of libraries was performed using Agencourt AMPure XP beads (Beckman Coulter, Brea, CA, USA), and their concentrations were measured with a high-sensitivity DNA chip on a Bioanalyzer 2100 (Agilent, Santa Clara, CA, USA). Multiplexed libraries were pooled into a single pool in equimolar concentrations and sequenced on Illumina HiSeq2500 with a 2 × 125 bp setup in the High Output mode. Libraries for modern samples were prepared according to New Zealand Genomics Limited (NZGL, Palmerston North, New Zealand) protocols for modern DNA and sequenced on Illumina HiSeq2500 with a 2 × 125 bp setup.

### 2.3. Data Processing

Bcl to Fastq conversion was performed using bcl2Fastq 1.8.3 from the CASAVA software suite. SeqPrep 1.1 [29] was used to trim adapters and merge paired-end reads, using default settings but with a minor modification in the source code, allowing us to choose the best quality scores of bases in the merged region instead of aggregating the scores following Palkopoulou et al. [3].

Sequencing reads were processed with BWA 0.7.13 [30] and SAMtools 1.3 [31] and then aligned to the reference mitogenome (GenBank accession No. NC_005931.1). For historical data, merged sequencing reads were mapped against the reference genome using the BWA aln algorithm and slightly modified default settings with deactivated seeding (-l 16,500), allowing more substitutions (-n 0.01) and allowing up to two gaps (-o 2). The BWA samse command was used to generate alignments. Reads mapping to the mitochondrial reference genome were extracted and processed in SAMtools, including converting the alignments from SAM format to BAM format, as well as coordinate sorting, indexing, and removing duplicates from the alignments.

For modern data, forward and reverse reads were mapped against the reference genome using the BWA mem command and then BAM files were generated using the SAMtools view command. Extraction, coordinate sorting, indexing, and duplicates removal for reads mapping to the mitochondrial genome was performed using SAMtools. For both historical and modern data, reads with mapping qualities below 20 were filtered out.

BAM files generated using SAMtools were then uploaded to Geneious^®^ 7.0.336 [32] and consensus sequences were called for positions with at least 3X coverage using a majority consensus rule, where ambiguous and low-coverage positions called as undetermined (N). Assembled sequences were then visually inspected to assess overall coverage across the 16,588 base pairs (bp) of the mitogenome and quality of the SNPs identified.

### 2.4. Changes in Genetic Diversity

Indices of genetic diversity including haplotypic diversity (Hd), nucleotide diversity (π) Tajima’s D [33] and Fu’s *F_S_* [34] were calculated in DnaSp v.5 [35] for historical and modern samples. Tajima’s D values > 0 suggest either a recent population bottleneck or balancing selection, while D < 0 indicates a population expansion or directional selection. Fu’s *F_S_* values < 0 suggest an excess of recent mutations (therefore an overabundance of rare alleles), characteristic of a recent population expansion, while positive Fu’s *F_S_* values indicate a deficiency of rare alleles, suggesting a population bottleneck or overdominant selection.

We then used a temporal approach to determine the loss of mitochondrial genetic diversity in kākāpō through time between historical (1847–1985) and modern samples (i.e., founders and F1s) using the R script TempNet v1.4. [36]. A median-joining haplotype network of all mitogenomes was also created in PopART [37].

### 2.5. Phylogenetic and Demographic Analyses

A phylogeny and demographic reconstruction were inferred in BEAST 1.8.044 [38] using the HKY+I substitution model, which was selected according to the Bayesian Information Criterion (BIC) in JModeltest2. We used a strict molecular clock with a uniform distribution and a lineage-specific substitution rate of 1–3 × 10^−8^ site^−1^ year^−1^ [39]. Three different tree models were tested: constant size, Bayesian Skyline and Bayesian Skyride. Constant size and Bayesian Skyride analyses were performed with default settings, while the number of groups in the Bayesian Skyline model was adjusted to 5 to avoid over-parametrization of the model [40]. To select the model with the best fit, we calculated the marginal likelihoods using path and stepping-stone sampling as implemented in BEAST 1.8.044 [41]. Bayes Factors were estimated as in Kass & Raftery [42] and the Bayesian Skyline model was selected and used for further analyses (Table S2). For all models, the Markov Chain Monte Carlo was set to run for 80 million generations, sampling every 5000th generation. Information from the sampled trees was summarized in Tree Annotator [38]. Tracer 1.647 [43] was used to compare the tested models, to verify convergence of the runs and to perform Bayesian Skyline reconstruction (using the default settings) estimating the female effective population size (*N*_ef_).

Finally, we tested for competing bottleneck models and estimated values of demographic parameters using an approximate Bayesian computation (ABC) approach [44] implemented in DIYABC 2.1.0 [45]. We first tested three general models: (1) ‘constant population size through time’; (2) ‘postglacial expansion’; (3) ‘postglacial expansion and recent bottleneck’. We used wide priors (Table S3) and a generation time of 25 years for kākāpō [46]. We then refined the model with the highest posterior probability (i.e., ‘postglacial expansion and recent bottleneck’, see Results), in order to distinguish between population bottlenecks associated with either Polynesian or European arrival. We designed three models: (3.1) a ‘Polynesian bottleneck’ describing a bottleneck occurring 20–30 generations ago, a period encompassing the arrival of Polynesians to New Zealand; (3.2) a ‘European bottleneck’ describing a more recent bottleneck occurring 1–10 generations ago; and finally, (3.3) a model of ‘Polynesian and European bottlenecks’ combining the bottlenecks of the previous two models. For each model, we simulated 1 million datasets and used the number of haplotypes, number of segregating sites, mean of pairwise differences, and Tajima’s *D* within each time period sample as summary statistics for the whole mtDNA. We calculated normalized euclidian distances between the observed dataset and each of the simulated datasets using the local linear regression method of Beaumont et al. [44]. We estimated the posterior probabilities of each scenario using a logistic regression approach [47,48]. We first estimated posterior probabilities between models (3.1) and (3.2) only and then among models (3.1), (3.2) and (3.3) together. We retained the 10,000 datasets (1%) with the smallest Euclidian distances to build posterior parameter distributions. Confidence in scenario choice was done by generating pseudo-observed datasets (pods) and by estimating the type I and type II errors as implemented in DIYABC 2.1.0. Additional details on scenario tested, parameter priors and mutation models can be found in Table S3.

### 2.6. Data Availability

BAM files for complete mitogenomes of 39 historical and 79 modern kākāpō are deposited in the European Nucleotide Archive (ENA), under the accession number (PRJEB25924).

## 3. Results

### 3.1. Genetic Diversity and Phylogeny

**Figure 1:**
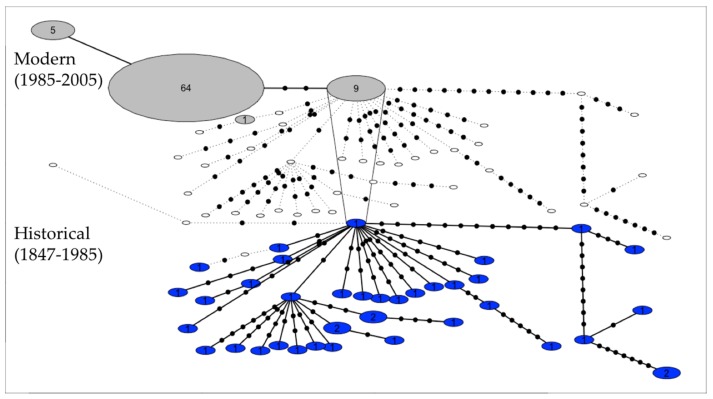
Temporal haplotype network displaying the change in mitogenome (16,588 bp) diversity through time for historical (in blue, bottom part; *n* = 39) and modern (in grey, top part; *n* = 79) kākāpō samples. Circles represent haplotypes and numbers represent sample sizes. Empty circles represent absent haplotypes for a given time period. Haplotypes found in multiple time periods are connected by vertical lines. Within each time period, black dots represent one mutation.

Genetic diversity was high among historical samples but very low among modern samples. We observed a ~30-fold and ~11-fold loss in haplotypic and nucleotide diversity respectively between historical and modern samples (Table 1). Four haplotypes were identified in the modern dataset and 36 haplotypes in the historical dataset. While modern haplotypes differed from historical ones by several mutations, all of these were synonymous in coding regions. There was only one haplotype in common across time periods (Figure 1). Tajima’s *D* and Fu’s *F*_S_ were both significantly negative in historical samples suggesting a recent population expansion (Table 1).

**Figure 2:**
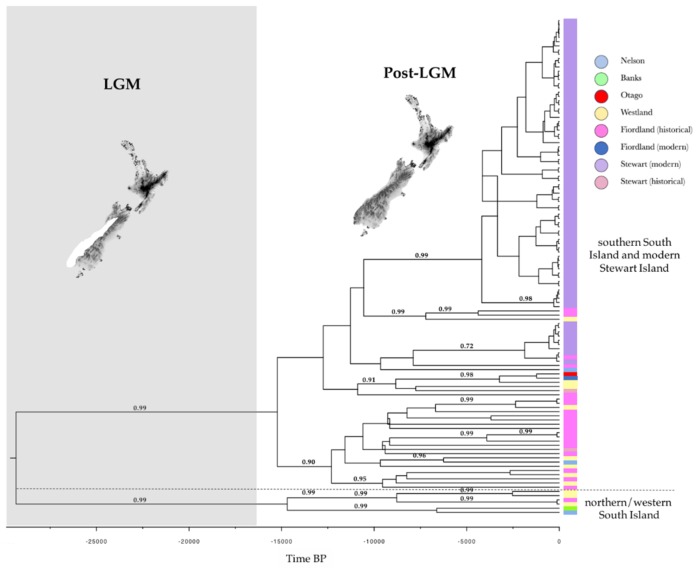
Bayesian phylogeny in kākāpō for full mitogenomes (16,588 bp). Timing of events was estimated assuming a substitution rate of 1–3 × 10^−8^ site^−1^ year^−1^ [39]. The *x*-axis is in calendar years before present and nodes with posterior probability larger than 0.7 are depicted. Maps show the extent of ice during (white) and after the last glacial maximum (LGM) (after [49]).

**Figure 3:**
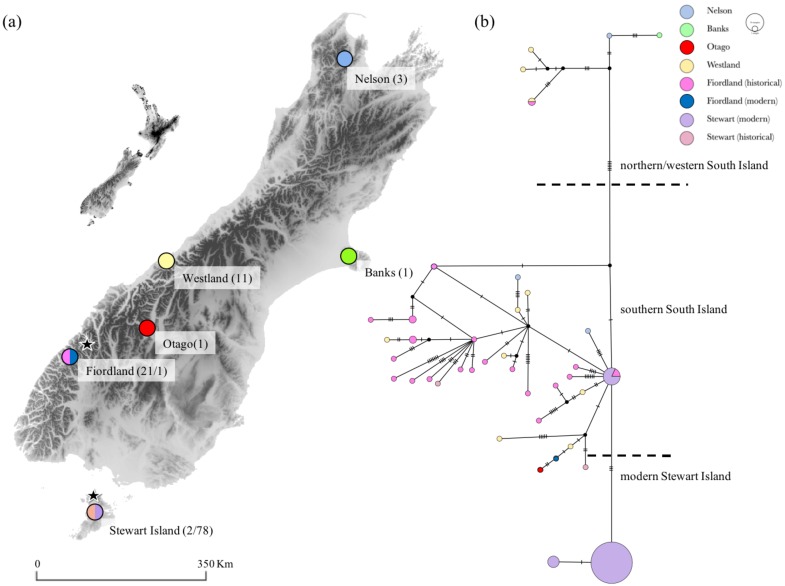
(**a**) Sampling locations for 39 historical and 79 modern kākāpō. Full circles represent sampling locations for historical specimens only and split circles refer to sampling locations for both historical specimens and modern birds. Numbers represent sample sizes for each location and dataset. Stars represent geographic origin of the founders. (**b**) Median-joining haplotype network for 118 kākāpō mitogenomes (16,588 bp). Geographical locations in the legend are listed from north to south.

The Bayesian phylogeny supported two distinct geographical clades: a ‘northern/western South Island’ clade and a ‘southern South Island and modern Stewart Island’ clade. Assuming a substitution rate of 1–3 × 10^−8^ site^−1^ year^−1^, the estimate for the divergence time of these two lineages was of 30 kya (95% highest probability density (HPD): 15,000–65,000) and 11 kya (95% HPD: 7500–22,000), respectively (Figure 2). The median-joining network showed additional geographical structuring with the ‘northern/western South Island’ lineage and the ‘modern Stewart Island’ lineage differing from a ‘southern South Island’ lineage by six and three mutational steps respectively (Figure 3). However, the two historical Stewart Island haplotypes were not clustering with modern Stewart Island ones and were three to seven mutational steps away from the most central Stewart Island haplotype (Figure 2 and Figure 3). Also, two out of three northern South Island samples (i.e., Nelson) were clustering with the main ‘southern South Island’ lineage (Figure 3).

### 3.2. Recent and Past Demography

**Figure 4:**
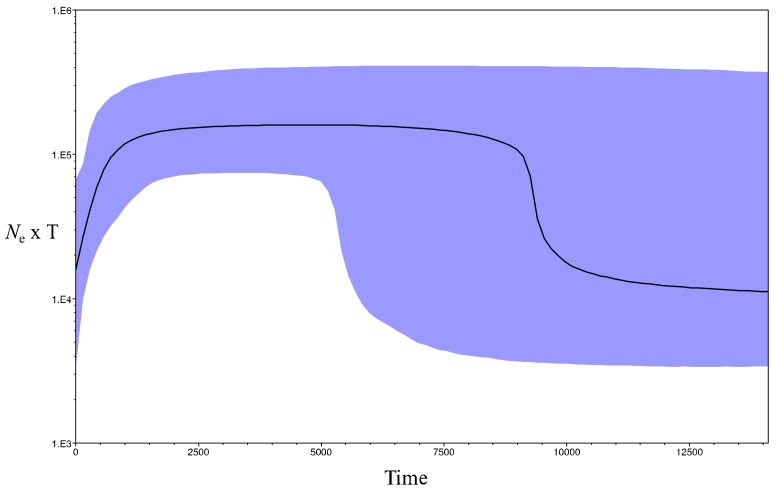
Bayesian Skyline plot (BSP) depicting population size change through time in kākāpō for full mitogenomes (16,588 bp). Timing of events was estimated assuming a substitution rate of 1–3 × 10^−8^ site^−1^ year^−1^ [39]. The x axis is in calendar years before present and y axis equals changes in effective population size (shown as the product of *N*_e_ and generation time T). The black line is the median estimate and the blue lines show the 95% highest posterior density intervals.

The Bayesian Skyline model had the highest log marginal likelihood among the compared models (Table S2). The Bayesian skyline plot supported an overall constant population size with some evidence for population expansion dating back some 10 kya and a recent population decline some 500 hundred years ago (Figure 4). Assuming a generation time of 25 years [46], the *N*_ef_ increased from c. 400 (95% HPD: 100–8000) to c. 4000 (95% HPD: 1200–8000) around 10 kya and dropped to c. 500 (95% HPD: 80–1200) some 500–1000 years ago.

When testing for demographic models using the ABC approach, a general model of ‘population expansion and recent bottleneck’ was strongly supported with a posterior probability of 99%. Post-glacial expansion was estimated at ~14,375 ya (95% HPD: 7975–14,700) (Table S3). Population size declined from a *N*_e-pre-human_ of 497,000 (95% HPD: 253,000–497,000) to a *N*_e-modern_ of 17.3 (95% HPD: 12.2–45.9). Bottleneck timing was estimated at ~120 ya (95% HPD: 57–145) (Table S3). When testing between two refined models of ‘European bottleneck’ and ‘Polynesian bottleneck’, the former obtained a posterior probability of 100% and Type I and II errors of 0.0 and 0.002 respectively. However, when comparing the three refined models of ‘European bottleneck’, ‘Polynesian bottleneck’ and ‘Polynesian and European bottlenecks’ together, their posterior probabilities were of 41%, 0% and 59%, respectively. Estimates of *N*_e-modern_ and *N*_e-pre-glaciation_ and t-bottleneck-Eu were consistent between the two models with highest posterior probabilities, with mode values ranging between 17.6–16.8, 3.9–5.91 × 10^3^, 4.47–4.24 respectively (Table 2). Type I and II errors for a model of ‘European bottleneck’ was 0.32 and 0.38 respectively, while these errors were 0.51 and 0.32 for a ‘Polynesian and European bottlenecks’ model. Determining which of these models was the most probable was therefore not possible.

## 4. Discussion

### 4.1. Genetic Diversity and Phylogeny

Using full mitogenomes we find high pre-European genetic diversity in kākāpō and strong support for a severe bottleneck coinciding with European arrival in New Zealand, which is consistent with Bergner et al. [22]. However, due to the geographically and temporally limited sampling (i.e., South Island samples from the 1800s to present day only), we could not capture most of the historical diversity of the species. We might thus expect to find even higher levels of genetic diversity if we obtained pre-human samples from the North Island (i.e., subfossil remains since no historical skins are available). Nevertheless, there is strong evidence for severe loss of haplotypic and nucleotide diversity in kākāpō in the last 200 years, which is consistent with the small number and reduced geographical representation of the founders of the modern population, as well as the documented demographic decline [9]. However, we did not find evidence for fixation of non-synonymous mutations with potential functional consequences in modern kākāpō.

The star-shape haplotype network, the significantly negative Tajima’s *D* and Fu’s *F*_S_ in historical samples, the increase in *N*_ef_ dating back 10–15 kya, and the divergence of distinct clades between 30 and 10 kya, are consistent with a population expansion at the end of the last glacial maximum (LGM), some ~14–18 kya [50,51,52,53]. In contrast with the results of Bergner et al. [22] that identified a single southern glacial refugium based on mtDNA control region, our results supported an additional northern glacial refugium. The Bayesian phylogeny identified two geographically distinct lineages albeit with very wide divergence date posteriors: a ‘northern/western South Island’ lineage and a ‘southern South Island’ lineage centred on Fiordland and Westland. In the median-joining network, these lineages were also obvious but the ‘southern South Island’ lineage comprised two haplotypes from the northern South Island (i.e., Nelson, Figure 3). This could suggest that this region may have acted as a single glacial refugium from where kākāpō recolonized its whole range at the end of the LGM. However, based on the present phylogenetic dichotomy and previous studies that have identified northern and southern refugia [54], the most parsimonious explanation for this placement is specimen mislabelling or authentication, which has been reported before with other historical samples [55]. Interestingly, these samples are currently held by the same museum, which further suggests that this mislabelling error may have occurred upon transfer of samples between collectors and later acquisition of the samples by the museum [55]. Kākāpō thus seems to be another example of phylogenetic dichotomy in the form of northern and southern lineages referred to as the ‘beech-gap’ hypothesis, as previously shown in a wide range of New Zealand taxa [54,56], such as avian species with limited dispersal capabilities (e.g., Rock Wren (*Xenicus gilviventris*) [57]; Kiwi (*Apteryx* sp.) [58]). This result therefore supports population persistence in forest refugia during the Pleistocene. However, this ‘northern/western South Island’ kākāpō lineage comprises Westland and Fiordland haploytpes, which suggests that a recolonization of Westland and Fiordland may have also occurred from a northern refugium along the western flank of the Alps.

It is also possible that the observed lineage divergence may be due to the underrepresentation of samples from the central and northern South Island. Obtaining historical samples from these regions is required in future studies to better investigate the existence of glacial refugia, confirm the ‘beech-gap’ hypothesis and to determine whether this split is the result of sampling bias. The potential of sampling bias raises the possibility that kākāpō either were restricted to a northern and southern refugium from where they rapidly expanded at the end of the LGM, or that they were continuously distributed on the eastern side of the South Island through the LGM. The latter scenario is possible given that kākāpō are considered successful habitat generalists, inhabiting forests, shrubland, grassland as well as transition zones between communities (e.g., forest and grasslands) [9], which were the predominant habitats on the eastern side of the South Island during glaciations [52,59,60]. In fact, a similar pattern was found in the closely-related kea (*Nestor notabilis*) [61] and in brown kiwi (*Apteryx* sp.) [62], which may both have survived at the margin of the glaciers in a habitat dominated by scrub and grasslands.

There has been speculation that the presence of kākāpō on Stewart Island may be the result of a recent introduction either by pre-European Polynesian settlers or by Europeans in the 1880s [19]. No fossil kākāpō remains have been found on Stewart Island [9,10], which might be further evidence that the Stewart Island kākāpō are relatively recent arrivals on the island. However, Powlesland et al. [24] suggested that the population of about 100 kākāpō discovered on Stewart Island in the mid 1970s was too large to have arisen from introductions by Europeans because of the species’ low fecundity. Our observation of a distinct mitochondrial lineage apparently endemic to Stewart Island suggests that kākāpō could have been present on this island for several thousands of years. The ‘modern Stewart Island’ linage comprised two of the most common modern Stewart Island haplotypes, which we estimate diverged some 11 kya (95% HPD: 7500–22,000). However, while this timing coincides with the geographical isolation of Stewart Island from the mainland following sea rise at the end of the LGM [50,52], the divergence of this lineage was rather shallow and comparable to the divergence of other haplotypes within the ‘southern South Island’ lineage. Moreover, we found a third modern Stewart Island haplotype that clustered with the mainland haplogroup and that was shared with historical Fiordland samples. These findings thus do not allow to exclude the hypotheses that pre-European Polynesian settlers or Europeans may have established a kākāpō population on Stewart Island or released kākāpō where a resident population already existed. This suggests that some of the contemporary birds descending from Stewart Island founders could actually represent some part of the mainland historical genetic diversity. Surprisingly, historical Stewart Island samples were placed on different branches of the mainland haplogroup and not on the ‘modern Stewart Island’ lineage. The most parsimonious explanation for this inconsistency in the placement of historical Stewart Island samples is that these sample may also be instances of museum specimen mislabelling or authentication [55], as both samples were held by the same museum.

### 4.2. Recent and Past Demography

Contrary to previous results based on microsatellite and mtDNA control region data [22], our Bayesian skyline plot detected a population bottleneck coinciding with Polynesian arrival in the South Island, while the ABC analysis suggested a combination of Polynesian and European bottlenecks was possible. However, the support for the latter model is only tentative for several reasons. First, there was considerable overlap between the prior distribution for the pre-European and pre-Polynesian effective population sizes, either suggesting that this decline was minimal or that it may have resulted from a methodological artefact. Secondly, Type I and Type II errors for this model, as well as the second most likely model of ‘European bottleneck’ were high when compared against each other, meaning that each of these models are equally likely to explain the observed data. Interestingly, when only comparing the ‘European bottleneck’ and ‘Polynesian bottleneck’ models, the former had 100% posterior probability and the latter 0%, suggesting that a recent population decline associated with Europeans arrival may have been the main driver of loss of genetic diversity in kākāpō. However, while there is no evidence for population decline prior to human arrival, the kākāpō range contracted substantially following Polynesian settlement c. 1280 CE [20], particularly in the North Island [9], probably in response to hunting as well as predation by introduced dogs. In the South Island, these population pressures, as well as widespread burning of forest, scrub and tussockland from c. 1300 CE, were probably also contributing factors in the initial decline of kākāpō [63]. Consequently, we might have expected some signature of population decline associated with Polynesian settlement.

One reason for the lack of signal associated with Polynesian settlement may be due to the technical difficulty of detecting sequential bottlenecks so close in time (i.e., ~20 generations apart), as well as the estimation of the bottlenecks’ magnitudes. Obtaining historical samples predating both Polynesians and Europeans settlement would thus be the only way to reliably estimate the timing and magnitude of such bottlenecks. In particular, the magnitude of the bottleneck is important to consider, because if the bottleneck associated with Polynesians was only moderate, its genetic signature may have been erased by the subsequent more severe bottleneck. While it is known that Polynesians hunted kākāpō [63,64], it seems that they only shifted to hunting kākāpō when larger prey, such as moa and seals, started to decline in the late 13th century [10,65]. Around this time Polynesians also switched to a more transient lifestyle, abandoning permanent settlements and adopting a more generalist hunting approach, where kākāpō may have been only seasonally hunted [64], which might have resulted in a moderate and localized decline of kākāpō in the South Island prior to Europeans. Conversely, the European bottleneck was quick, severe and widespread [9] with strong evidence of flightless species being the most severely impacted by the introduction of new mammalian predators [66,67]. Therefore, while we cannot discount effects of Polynesians activities on the genetic diversity of kākāpō, the species may not have been significantly affected in the South Island prior to European arrival [22]. This in turn reinforces the hypothesis that the introduction of predators by Europeans may have been the most important cause of severe decline and ensuing near-extinction of kākāpō on the South Island.

Despite the pre-European abundance of kākāpō, the species’ low reproductive rates, intermittent breeding (occurring at two to seven year intervals) linked to podocarp tree masting, and lek-breeding behaviour [9,23,68] may have accelerated the loss of genetic diversity following the severe population decline. This is in stark contrast with the closely-related kea (*Nestor notabilis*) which is apparently more resilient to population decline and more resistant to loss of genetic diversity [69] owing to its monogamous reproductive behaviour and annual reproduction.

## 5. Conclusions

Our results highlight the use of temporal approaches to examine the genomic consequences of population declines. Recent studies on woolly mammoths showed an increase in deleterious mutation in both mitogenomes [7] and nuclear genomes [6]. Here, we did not find evidence for fixation of deleterious mutations in mtDNA coding regions of modern kākāpō. Nevertheless, the extreme loss of genetic diversity observed in kākāpō still suggests that substantial amounts of functional nuclear diversity have been lost and that deleterious mutations may have accumulated, which might have important consequences for the fitness and long-term persistence of the species. Comparing full historical and modern nuclear genomes will provide an understanding of the increase in inbreeding and accumulation of deleterious mutations and their effect on fitness of the remaining kākāpō.

## Figures and Tables

**Table 1:** Overall genetic diversity indices for 118 historical and modern kākāpō mitogenomes (16,588 bp) showing the sample size (*n*), number of observed haplotypes (h), number of polymorphic sites (S), haplotypic diversity (Hd), nucleotide diversity (π) with their respective standard error (SE) in brackets and Tajima’s D and Fu’s *F*_S_.

Time Period	*n*	h	S	Hd	π	D	*F*_S_
Modern	79	4	7	0.0331 (0.024)	0.00005 (0.00002)	−0.944	0.859
Historical	39	36	100	0.996 (0.0064)	0.00059 (0.00006)	−2.16 **	−24.48 **

** Significant at *p* < 0.02.

**Table 2:** Prior and posterior distributions of parameters for refined models of (a) ‘European bottleneck’ and (b) ‘European and Polynesian bottlenecks’. Timing of events corresponds to number of generations and assumes a kākāpō generation time of 25 years [46].

**(a)**	**Parameters**	**Prior**	**Posterior Mode**	**5% HPD**	**95% HPD**
	*N*_e-modern_	Uniform (1–200)	17.6	7.65	96.5
	*N*_e-pre-European_	Uniform (5 × 10^3^–6 × 10^5^)	5.67 × 10^5^	1.80 × 10^5^	5.86 × 10^5^
	*N*_e-pre-glaciation_	Uniform (10^3^–3 × 10^5^)	3.90 × 10^3^	2.61 × 10^3^	5.75 × 10^4^
	t-bottleneck-Eu	Uniform (1–10)	4.47	2.44	5.56
	t-post-glaciation	Uniform (300–600)	600	354	600
	μ rate	Uniform (10^−8^–10^−7^)	1 × 10^−7^	6.45 × 10^−8^	1 × 10^−7^
**(b)**					
	*N*_e-modern_	Uniform (1–200)	16.8	6.38	88.6
	*N*_e-pre-human_	Uniform (5 × 10^3^–6 × 10^5^)	5.76 × 10^5^	2.28 × 10^5^	5.90 × 10^5^
	*N*_e-pre-European_	Uniform (5 × 10^3^–6 × 10^5^)	2.66 × 10^5^	4.62 × 10^4^	5.17 × 10^5^
	*N*_e-pre-glaciation_	Uniform (10^3^–3 × 10^5^)	5.91 × 10^3^	2.87 × 10^3^	6.22 × 10^4^
	t-bottleneck-Pol	Uniform (20–30)	20.1	20	30
	t-bottleneck-Eu	Uniform (1–10)	4.24	2.32	5.42
	t-post-glaciation	Uniform (300–600)	600	345	594
	μ rate	Uniform (10^−8^–10^−7^)	1 × 10^−7^	6.25 × 10^−8^	1 × 10^−7^

Conditions: (a) *N*_e-pre-European_ > *N*_e-pre-glaciation_; (b): *N*_e-pre-human_ > *N*_e-pre-glaciation_; *N*_e-pre-human_ > *N*_e-pre-European_.

## Supplementary Materials

The following are available online at http://www.mdpi.com/2073-4425/9/4/220/s1.
