# Supplementary material for: Full Mitogenomes in the Critically Endangered Kākāpō Reveal Major Post-Glacial and Anthropogenic Effects on Neutral Genetic Diversity
Source: Genes (Basel). 2018 Apr 19;9(4):220. doi: 10.3390/genes9040220 (PMC5924562; doi:10.3390/genes9040220)
Supplement: Supplementary file 1 [file genes-09-00220-s001.pdf]

genes

Full mitogenomes in the critically endangered kākāpō reveal major post-glacial and anthropogenic effects on neutral genetic diversity

Nicolas Dussex [1,2,*], Johanna von Seth [1,3], Bruce C. Robertson [2] and Love Dalén [1]

1 Department of Bioinformatics and Genetics, Swedish Museum of Natural History, SE-10405 Stockholm, Sweden; johanna.vonseth@nrm.se (J.v.S.); Love.Dalen@nrm.se (L.D.)
2 Department of Zoology, University of Otago, Great King Street, Dunedin 9016, New Zealand; bruce.robertson@otago.ac.nz (B.C.R)
3 Department of Zoology, Stockholm University, SE-10691, Stockholm, Sweden
* Correspondence: nicolas.dussex@gmail.com (N. D.); Tel.: +46-700-317-026

Table S1. Sampling location, date and museum information for the 39 historical kākāpō specimens used in this study.

**Museum**	**Museum no.**	**Locality**	**Region**	**Collection date**	**Lab code**	**Used in this stidy**	**Used inBergner et al (2016)**
American Museum of Natural History (USA)	623843	Otago	Otago	<1985	AMNH1	x	x
American Museum of Natural History (USA)	623841	Milford Sound	Fiordland	<1985	AMNH2	x	x
American Museum of Natural History (USA)	623839	Milford Sound	Fiordland	<1985	AMNH3	x	x
American Museum of Natural History (USA)	623836	Lake Te Anau, west shore	Fiordland	<1985	AMNH4		x
American Museum of Natural History (USA)	623836	Lake Te Anau, west shore	Fiordland	<1985	AMNH5	x	x
American Museum of Natural History (USA)	623835	Lake Te Anau, west shore	Fiordland	<1985	AMNH6	x	x
American Museum of Natural History (USA)	623840	Milford Sound	Fiordland	<1985	AMNH7	x	x
American Museum of Natural History (USA)	623837	West Coast South Island	Westland	<1985	AMNH8	x	x
American Museum of Natural History (USA)	623838	Jackson's Bay	Westland	<1985	AMNH9	x	x
Auckland Museum (NZ)	AU41.11	Pyke's Creek, Upper Hollyford River	Fiordland	1894	AUC1		x
Auckland Museum (NZ)	AU41.12	Lake McKerrow	Fiordland	1894	AUC2	x	x
Auckland Museum (NZ)	AU41.19	Fiordland	Fiordland	<1985	AUC3	x	x
Australian Museum	O30428	Nelson	Nelson	1912	AUS1	x	x
Australian Museum	O37315	Nelson	Nelson	1922	AUS3	x	x
Australian Museum	O37317	Jackson's Bay	Westland	1922	AUS4	x	x
Canterbury Museum (NZ)	16595	Clinton Valley Milford Sound	Fiordland	1893	CAN1	x	x
Canterbury Museum (NZ)	2059	Preservation Inlet	Fiordland	1898	CAN2	x	x
Canterbury Museum (NZ)	2922	West Coast, SI	Westland	<1985	CAN3	x	x
Canterbury Museum (NZ)	2923	West Coast South Island	Westland	<1985	CAN4	x	x
Canterbury Museum (NZ)	2924	West Coast, SI	Westland	<1985	CAN5	x	x
Canterbury Museum (NZ)	5186	Banks Peninsula (donated by school located there)	Banks	<1985	CAN6	x	x
Museum of Comparative Zoology, Harvard (USA)	170073	Arawata Mts	Westland	<1985	MCZ1	x	x
Museum of Comparative Zoology, Harvard (USA)	170074	Western Slopes, Southern Alps	Westland	<1985	MCZ2	x	x
Museum of Comparative Zoology, Harvard (USA)	170072	Arawata Mts	Westland	<1985	MCZ3	x	x
Museum of Comparative Zoology, Harvard (USA)	148166	Western slope Mt Cook	Westland	<1985	MCZ4	x	x
Museum of Natural History Vienna (Austria)	45.794	western South island	Westland		VM1		x
Museum of Natural History Vienna (Austria)	12.224	Alps, Dusky Bay, SI	Fiordland	1884	VM2		x
Museum of Natural History Vienna (Austria)	12.218	Alps, Dusky Bay, SI	Fiordland	1884	VM10	x	x
Museum of Natural History Vienna (Austria)	12.221	Alps, Dusky Bay, SI	Fiordland	1884	VM11	x	x
Museum of Natural History Vienna (Austria)	12.217	Alps, Dusky Sound, SI	Fiordland	1884	VM12	x	x
Museum of Natural History Vienna (Austria)	12.223	Alps, Dusky Bay, SI	Fiordland	1884	VM13	x	x
Museum of Natural History Vienna (Austria)	12.216	Alps, Dusky Sound, SI	Fiordland	1884	VM14	x	x
Museum of Natural History Vienna (Austria)	12.228	Alps, Dusky Bay, SI	Fiordland	1884	VM3	x	x
Museum of Natural History Vienna (Austria)	50.441	Alps, Dusky Sound, SI	Fiordland	1884	VM4	x	x
Museum of Natural History Vienna (Austria)	50.442	Alps, Dusky Sound, SI	Fiordland	1884	VM5	x	x
Museum of Natural History Vienna (Austria)	12.229	Alps, Dusky Bay, SI	Fiordland	1884	VM6		x
Museum of Natural History Vienna (Austria)	50.444	?	Fiordland		VM7		x
Museum of Natural History Vienna (Austria)	12.222	Alps, Dusky Sound, SI	Fiordland	1884	VM8		x
Museum of Natural History Vienna (Austria)	12.22	Alps, Dusky Bay, SI	Fiordland	1884	VM9		x
National Museum of Natural History Leiden (Netherlands)	RMNH.AVES.166785	Stewart Is	Stewart	1847	LEI1	x	x
National Museum of Natural History Leiden (Netherlands)	RMNH.AVES.166786	Stewart Is	Stewart	1847	LEI2	x	x
Otago Museum (NZ)	390	Fiordland	Fiordland	<1985	OTA1		x
Otago Museum (NZ)	5744	Martin's Bay, South of Haast	Fiordland	<1985	OTA2	x	x
Otago Museum (NZ)	8947	Resolution Island?	Fiordland	<1985	OTA3	x	x
Te Papa (NZ)	19010	Jackson's Bay	Westland	<1922	TEP10	x	x
Te Papa (NZ)	1365	Goulden Downs	Nelson	1924	TEP11	x	x
Te Papa (NZ)	11607	Tutoko Valley, Fiordland	Fiordland	1961	TEP3	x	x
Te Papa (NZ)	22705	Esperance Valley, Fiordland	Fiordland	1974	TEP4	x	x
Te Papa (NZ)	11560	Tutoko Valley, Fiordland	Fiordland	1961	TEP2		
Te Papa (NZ)	9487	Cheddar Valley, Fiordland	Fiordland	1961	TEP5		x
Te Papa (NZ)	13440	Sinbad Valley, Milford Sd	Fiordland	1967	TEP7		x
Te Papa (NZ)	1985	Dusky Sound	Fiordland	1884	TEP8		x
Te Papa (NZ)	1988	Nelson	Nelson	1897	TEP9		x

Table S2. Log Marginal Likelihood for the three models of constant size, Bayesian Skyline and Bayesian Skyride and Bayes factor values for the preferred model (i.e. Bayesian Skyline) relative to the other models. The log Bayes Factor corresponds to the difference between the preferred model and the alternative model.

Model	Stepping-stone Sampling		Path Sampling	
	Log Marginal Likelihood	Log Bayes Factor	Log Marginal Likelihood	Log Bayes Factor
Constant	-23862.96	11.90	-23861.51	13.10
Skyline	-23851.06	*	-23848.41	*
Skyride	-23886.02	34.96	-23883.52	35.11

Table S3. Prior and posteriors distribution of parameters for a general model of 'postglacial expansion and recent bottleneck'. Timing of events corresponds to number of generations and assuming a generation time of 25 years for kākāpō [47].

Parameters	Prior	Posterior mode	5% HPD	95% HPD
$N_{\text{e-modern}}$	Uniform (10-500)	17.3	12.2	45.9
$N_{\text{e-pre-human}}$	Uniform (10^3-$6x10^5$)	$4.97x10^5$	$2.53x10^5$	$4.97x10^5$
$N_{\text{e-pre-glaciation}}$	Uniform (10^3-$6x10^5$)	$1.07x10^5$	$7.36x10^4$	$3.55x10^5$
t-bottleneck	Uniform (1-50)	4.76	2.32	5.77
t-post-glaciation	Uniform (300-600)	575	319	588
μ rate	Uniform (10^{-8}-10^{-7})	$3x10^{-7}$	$1.61x10^{-8}$	$3x10^{-7}$

Conditions: $N_{\text{e-pre-human}} > N_{\text{e-pre-glaciation}}$
